# Supplementary material for: A Critical View of the Application of the APEX Software (Aqueous Photochemistry of Environmentally-Occurring Xenobiotics) to Predict Photoreaction Kinetics in Surface Freshwaters
Source: Molecules. 2019 Dec 18;25(1):9. doi: 10.3390/molecules25010009 (PMC7017383; doi:10.3390/molecules25010009)
Supplement: Supplementary file 1 [file molecules-25-00009-s001.zip › 3.2.4_gcc-4.4.0.zip!/3.2.4_gcc-4.4.0/3.2.4_gcc-4.4.0/doc/octave/html/interpreter/Stair-group.html]

Stair group - Untitled


Next: Stem Series,
Previous: Scatter group,
Up: Object Groups


---

##### 15.2.8.9 Stair group

Stair series objects are created by the `stair` function. Each
`hggroup` element of the series contains a single line object as a
child representing the stair. The properties of the stair series are

`color`: The RGB color or color name of the line objects of the stairs. See Colors. `linewidth` `linestyle`: The line width and style of the line objects of the stairs. See Line Styles. `marker` `markeredgecolor` `markerfacecolor` `markersize`: The line and fill color of the markers on the stairs. See Colors. `xdata` `ydata`: The original x and y data of the stairs. `xdatasource` `ydatasource`: Data source variables.
